# An uncut copy of *Scleromyceti Sueciae*: lost and then found

**DOI:** 10.1186/s40529-024-00414-2

**Published:** 2024-03-01

**Authors:** Roy Watling

**Affiliations:** 26 Blinkbonny Av. Edinburgh EH4 3HU, Scotland, UK

**Keywords:** Fire, Library-trawl, Discovery

## Abstract

**Background:**

A copy of *Scleromyceti Sueciae*, a work on which the nomenclature of many fungi is based was known to occur in Scotland’s Glasgow University Botany Department but the buildings were devastated by fire in 2001 and the whereabouts of this important work, if it existed, was lost. Its re-finding is reported herein.

**Results:**

The Glasgow copy of *Scleromyceti Sueciae*, an uncut first edition of Fries’ work, was located in the Glasgow Museums in its original cabinet being transferred there years before the fire and its specimens being now databased. It is one of the few existing uncut copies of this important scientific work and one of the best-preserved copies of the first edition.

**Conclusion:**

The discovery of this first edition of *Scleromyceti Sueciae* emphasizes the significance to reserve special conservation for important collections by early mycologists. It also allows interested mycologists world-wide to know of the existence in Glasgow of an uncut, first edition copy.

## Background

I had the honour to meet Jack Rogers both in Edinburgh and Liverpool, and at meetings and conferences home and abroad. He always instigated lively discussions and was always enthusiastic to include students therein. He and Belle were gracious hosts when I stayed with them in Pullman. It was a pleasure to host Jack, Belle and the family on their visit to Scotland and later to take Jack to Sunderland Museum, UK, to see James Bolton’s specimens of *Sphaeria concentrica* Bolton collected by Edward Robson in Northern England. These were the very same specimens described and figured by Bolton and on which the name *Daldinia concentrica* (Bolton) Ces. & De Not. is based (Rogers et al. 1999).

The *Daldinia* Ces. & De Not. find had stimulated an interest in old collections and during a review of Edinburgh’s Royal Botanic Garden mycological collections (fungarium) it became apparent that housed therein was a large number of Elias Fries’ collections from his *Scleromyceti Sueciae.* It is this same Fries whose *Systema Mycologicum* (Fries 1821, 1822–1823, 1829–1832) became, for most fungal groups, the starting point for nomenclature; so material distributed by Fries is of the utmost importance.

The material in Edinburgh had been cut from its original folders, as had many other copies located in Europe, each species being filed in their respective fungaria under the current name of the fungus. Prof. C. L. Shear (Beltsville, Maryland, USA), who visited Uppsala, Sweden, in 1905, whilst making a special study of Fries’ publication, found eight fascicles made up of Decades I–IV. He considered that the Edinburgh material on examination was part of a second edition of Fries’ work, not as important, but of considerable note.

However, Stephen Hutchinson, mycologist in the Department of Botany in Glasgow, produced a brief account of the discovery of an uncut, first edition of the *Scleromyceti* (Hutchinson 1964) in his department. At the request of the then professor he indicated that if anyone wished to see it, they would require permission as it was kept in the professor’s private rooms in a special cabinet (- little did we know that this was exceedingly fortuitous). It appears to be one of the few existing copies of *Scleromyceti*, which, although lacking one fascicle, is almost complete and one of the best-preserved copies of the first edition of this important scientific work.

In Holm and Nannfeldt (1962), there is a page (pg. 21) of handwriting provided by Shear that shows most of the higher numbers of the second edition had been labelled by a ‘secretary’, who later was found to be called Wahlenberg. The second edition appears to have been offered for sale in autumn of 1834 and is not as complete as Fries did not have printed labels for the collections or he used old ones. It appears to have been prepared (in haste?) before Fries left Lund for a position in Uppsala. Holm and Nannfeldt (1962) published an appendix to the Title pages for Fascicles 2–4, a Check list (pg. 25–39) and a commentary on the latter list (pg. 39–51), as well as the correspondence with Shear (1962) as an attachment; indices are rarely seen and there is some doubt as to whether Fascicle 9 was ever produced. The second edition, as exemplified by the Edinburgh material, was apparently simpler, the specimens being glued to loose pieces of paper 10 × 8 cm. Specimens 1–340 apparently were issued with the intention of duplicating the original edition; 341–460 had not been distributed before and appear to be also labelled by Wahlenberg. Holm and Nannfeldt (1962) were unaware of the existence of a second edition and it was only through Shear’s investigation and diligence that such an edition became known. In addition, all three authors were unaware that a first edition was known in Scotland!

**Fig. 1:**
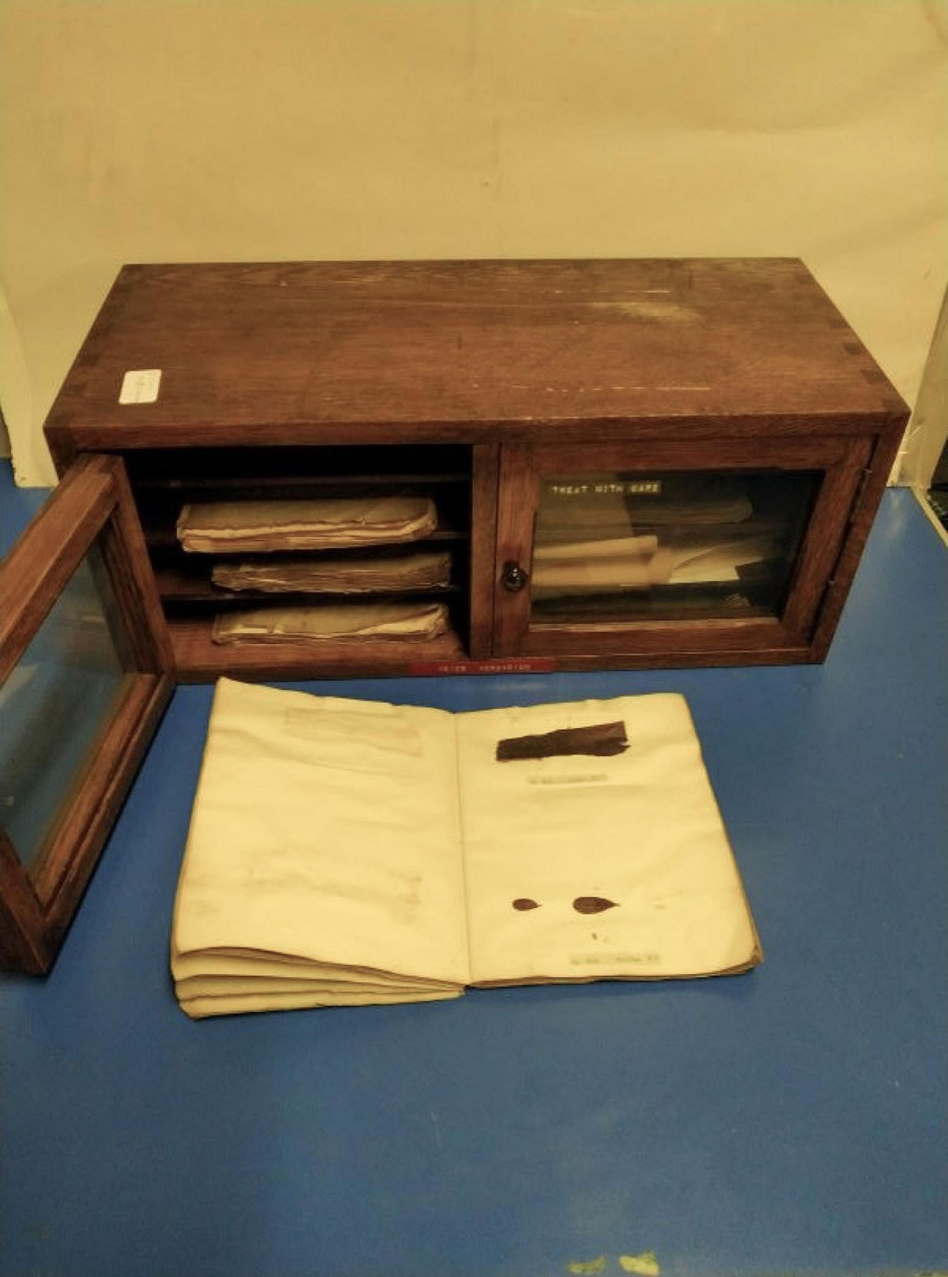
Complete cabinet of Fries herbarium

Possibly by a chance reading of Holm, Nannfeldt and Shears’ investigations, Prof. John Walton, the then Regius Keeper of Botany at Glasgow University, recognized the importance of the find when he located, amongst artifacts of previous chairs of the Glasgow Botany Department, a copy of *Scleromyceti*. It had been possibly purchased by the successor of John Hutton Balfour, Regius Chair of Botany at Glasgow University (1841–1845), Prof. George Walker Arnott (1845–1867), when he retired. Balfour already had made strong connections with Sweden having been awarded the King Gustaf Medal. The publication was in a wooden case with eight shelves (Fig. 1).

## Methods

### Fungal material

The Glasgow copy of *Scleromyceti* Sueciae was studied. This is a first edition of a work by Elias Fries, a Swedish mycologist, on which the nomenclature of many fungi is based. It is one of the few existing and uncut copies of this important scientific work, and it is kept in a specially crafted cabinet in the Glasgow Museums (Fig. 1).

### People contacted for searching the uncut copy of ***Scleromyceti Sueciae***

Richard Weddle, the manager of the local biological records centre at the Resources Department of the Glasgow Council, was contacted to ask if the copy was deposited there. Nicki Russel, from the University of Glasgow Archives and Special Collections, was contacted to check the catalogues at the library and the Hillhead Street deposit. Mike Blatt, the current Regius Keeper of Botany at Glasgow University, was contacted to ask if the copy was among the books that were saved from the fire in 2001. Giovanna Vitelli, The Hunterian Museum, was contacted to see if the copy was transferred there along with the mycological collection. Keith Watson, the curator of the Glasgow Museums, was contacted and confirmed the location and the condition of the copy.

## Results and discussion

### Contents of the glasgow copy

**Fig. 2:**
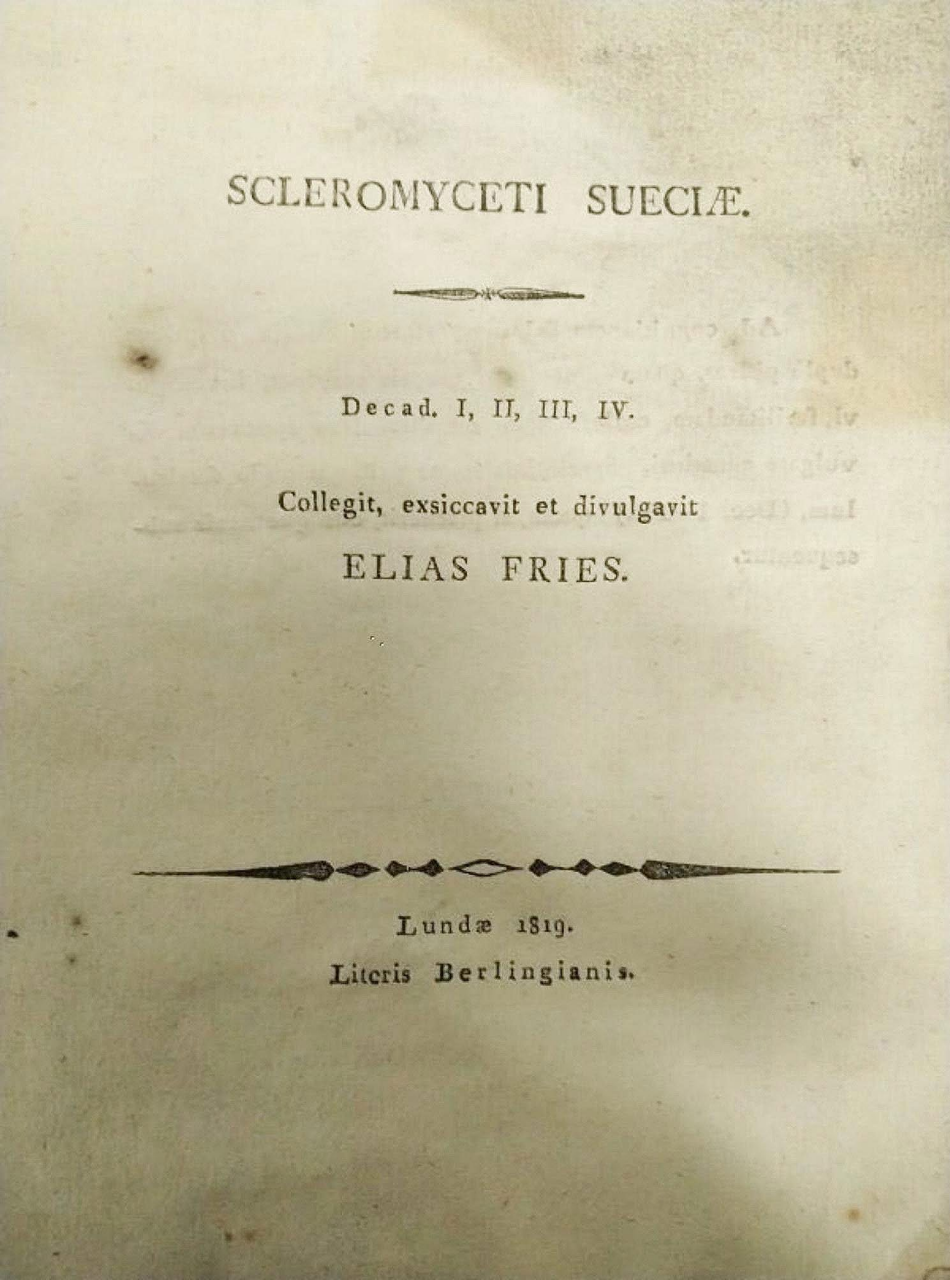
Title page to *Scleromyceti Sueciae*

**Fig. 3:**
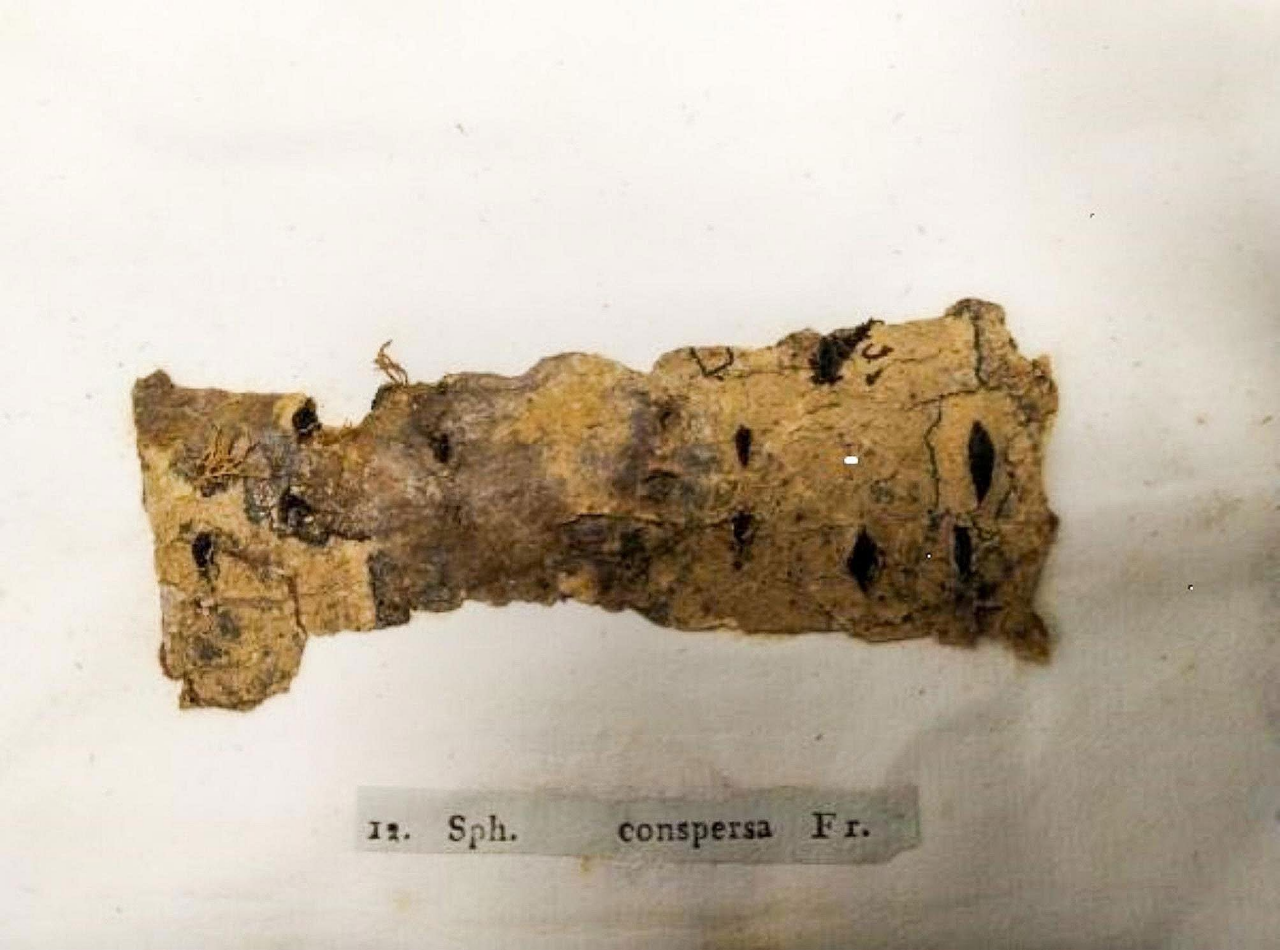
Example of specimen in *Scleromyceti*. *Sph*(*aeria*) *conspersa*, now *Tympanis conspersa* (Tympaidaceae)

The Glasgow copy is composed of sheets approximately 21 × 16 cm and bound with notes and indices in original grey cover (Figs. 1 and 2). It is in its own fashioned cabinet and consists of the following Fascicles: 1 Decades I − IV 1819 (Figs. 2 and 3); 2 Decades V − VII 1820; 3 Decades VIII − XI 1820; 4 Decades XII − XIV 1820; 5 Decades XV − XVIII 1821; 6 Decades XIX − XXII 1821; 8 Decades XXVIII − XXX 1822. Fascicle 7 is missing and was not found on the original discovery. There have been several moves of the Botany Department since the time of George Walker Arnott, so Fascicle 7 may be permanently lost or have never been sent to Glasgow.

There are some differences in the Glasgow collection and the Check-list produced by Holm and Nannfeldt (1962) who already indicated that there are some misidentifications due to changes in what is understood currently to be under that name, some collections are different in what is indicated as being the same in each issue and some are immature. Hutchinson (1964) found that some specimens were missing and indicated that No. 36 *Sphaeria complanata* now *Calophoma complanata*, Didymellaceae; No. 88 *Lophium excipuliformis*, Mytillinidiaceae of uncertain position; No. 120 *Sphaeria pulvis-pyrius* which may never have been issued or is not to be found in all issues; it is now *Melanomma pulvis-pyrius*, Melanomataceae; No 185 *Sphaeria microstoma* now *Cytospora populina*, Valsaceae; No. 274 *Sphaeronema pyriforme* a species not recognized in the Index of Fungi; and No. 88 have all been removed or have fallen off as there is a glue spot where the material should be. No. 26 *Sphaeria* (*Crer*.) with cut across label in Fascicle index is given as sp. *Gnomon* Tul. but amended by Fries (?) to *G. tubaeformis* now in *Gnomoniella.* Vol. 3 of Systema Mycologicum (Fries 1829–1832) was never issued in the Glasgow copy.

### The search

Even though he was a palaeobotanist by training, Prof. Walton realized the significance of this collection and that specimens in copies of this work formed part of the descriptions on which many of the species are based. This made it one of the most important mycological collections held in Scotland. Sadly since that date the Bower building of the Botany Department, as it was then known, in 2001 suffered a devastating fire, which destroyed most of the structures of the department and their effects.

So, what had become of *Scleromyceti* and could it have survived the blaze as it was in a sealed container? If the latter no-one knew where it might be. My immediate thoughts that as mycology had since disappeared from the Glasgow University curriculum I contacted Richard Weddle the manager of the local biological records centre at the Resources Department of the Glasgow Council, thinking it might have been deposited there. Richard reported to me that it was not deposited there and did not know where it might be, if it had been saved. He suggested I should contact Nicki Russel (University of Glasgow, Archives and Special Collections). She checked the catalogues at the library at their deposit at Hillhead Street, Glasgow, and the item had not come to them. Nor was it in the Glasgow main library according to the Research Associate Maggie Reilly. Prof. Mike Blatt, the current Regius Keeper of Botany reported that some books had been saved from the Bower building during the fire of 2001 but *Scleromyceti* was not known to be among them. However, the wife of Prof. Malcolm Wilkins, another Regius Professor of the Department, who had taken over the maintenance of the mycological specimens, told me that she thought that it had not been damaged. Some books and manuscripts were held in a separate area of the department where the professors had their room so it was possible, it may have gone elsewhere. Nicki kindly suggested that I should contact the Hunterian Museum where the mycological collection may have gone. However, with the help of Giovanna Vitelli it was ascertained that there was no record of the publication there either. With even more enquiries by Richard the case was located in the Glasgow Museums and its curator, Keith Watson, kindly sent to me a photograph of the specially crafted cabinet in which the specimens are still safely kept. During the 1990s the Botany Department’s large herbarium had moved from the Bower Building to the Graham Kerr Building (formerly the Zoology Department) as part of a restructuring of life sciences. The Fries cabinet was apparently transferred to the Glasgow Museums along with other associated material at this time, although its significance was not then appreciated. It looks as if the Glasgow copy of *Scleromyceti* described by Hutchinson (1964) had indeed been saved from the fire because the room in which it had been kept for its protections, as suggested above, was not damaged by the fire being separately housed from the main building.

### Successful ending

Holm and Nannfeldt (1962) say ‘mycologists who have copies of *Scleromyceti* in their charge should try to find out the complete composition of their copy and encourage any author to relook at their copy to expand the present study where-ever-possible’. It was therefore considered that a published note was topical, especially as Jack Rogers spent his illustrious career studying the sphaeriaceous fungi, many of which are contained in the *Scleromyceti* and referred frequently to type specimens and descriptions. It would also allow interested authorities world-wide to know of the existence in Glasgow of an uncut, first edition copy. Keith Watson has subsequently databased all the specimens in the Decades under their original names and this will be available on the Glasgow Museum’s website.

## Conclusion

The Glasgow copy of *Scleromyceti Sueciae* is an uncut, first edition of Fries’ work, being of great importance for the nomenclature and taxonomy of many fungi. It was thought to be lost in a fire, but it was later found in the Glasgow Museums. The discovery of this first edition of *Scleromyceti Sueciae* emphasizes the significance of reserving special conservation for important collections by early mycologists. It also allows interested mycologists world-wide to know of the existence in Glasgow of an uncut copy of *Scleromyceti Sueciae*.

## Data Availability

The studied material is kept at Glasgow Museums, Scotland, UK.
